# Histone Acetylation and CREB Binding Protein Are Required for Neuronal Resistance against Ischemic Injury

**DOI:** 10.1371/journal.pone.0095465

**Published:** 2014-04-18

**Authors:** Ferah Yildirim, Shengbo Ji, Golo Kronenberg, Angel Barco, Roman Olivares, Eva Benito, Ulrich Dirnagl, Karen Gertz, Matthias Endres, Christoph Harms, Andreas Meisel

**Affiliations:** 1 Department of Experimental Neurology, Center for Stroke Research Berlin (CSB) and Klinik und Hochschulambulanz für Neurologie, Charité–Universitätsmedizin Berlin, Berlin, Germany; 2 Instituto de Neurociencias de Alicante (Universidad Miguel Hernandez-Consejo Superior de Investigaciones Cientificas), Campus de Sant Joan, Sant Joan d'Alacant, Alicante, Spain; 3 Klinik und Poliklinik für Psychiatrie, Campus Mitte, Charité–Universitätsmedizin Berlin, Berlin, Germany; 4 ExcellenceCluster NeuroCure, Charité–Universitätsmedizin Berlin, Berlin, Germany; Julius-Maximilians-Universität Würzburg, Germany

## Abstract

Epigenetic transcriptional regulation by histone acetylation depends on the balance between histone acetyltransferase (HAT) and deacetylase activities (HDAC). Inhibition of HDAC activity provides neuroprotection, indicating that the outcome of cerebral ischemia depends crucially on the acetylation status of histones. In the present study, we characterized the changes in histone acetylation levels in ischemia models of focal cerebral ischemia and identified cAMP-response element binding protein (CREB)–binding protein (CBP) as a crucial factor in the susceptibility of neurons to ischemic stress. Both neuron-specific RNA interference and neurons derived from CBP heterozygous knockout mice showed increased damage after oxygen-glucose deprivation (OGD) *in vitro*. Furthermore, we demonstrated that ischemic preconditioning by a short (5 min) subthreshold occlusion of the middle cerebral artery (MCA), followed 24 h afterwards by a 30 min occlusion of the MCA, increased histone acetylation levels *in vivo*. Ischemic preconditioning enhanced CBP recruitment and histone acetylation at the promoter of the neuroprotective gene gelsolin leading to increased gelsolin expression in neurons. Inhibition of CBP's HAT activity attenuated neuronal ischemic preconditioning. Taken together, our findings suggest that the levels of CBP and histone acetylation determine stroke outcome and are crucially associated with the induction of an ischemia-resistant state in neurons.

## Introduction

Histone acetylation is an important epigenetic mechanism for transcriptional control and its levels are regulated by the activities of histone acetyltransferases (HATs) and deacetylases (HDACs). The HAT–HDAC system is also involved in the modulation of other chromatin-associated processes such as replication, site-specific recombination and DNA repair [1]. Aberrant histone acetylation and/or impaired function of the histone acetylation machinery have been linked to pathogenic progression in numerous neurological conditions including neurodegeneration [2], [3], [4]. HDAC inhibitors have been used consistently and successfully in an array of neurological disease models including poly-glutamine toxicity, spinal muscular atrophy, intracerebral hemorrhage and cerebral ischemia [5], [6], [7], [8], [9], [10], [11]. CBP is a transcriptional co-activator with HAT activity that was shown to be important for long-term memory processes, which depend on *de novo* gene expression [12], [13], [14]. Mutations in its gene (*Crebbp*) underlie most cases of Rubinstein-Taybi syndrome, a rare neurodevelopmental disorder with mental retardation [15]. In contrast to several chronic neurodegenerative disease models [16], [17], [18], [19], the role of CBP and histone acetylation in acute neurological diseases like cerebral ischemia are poorly understood.

We have previously shown that the HDAC inhibitor Trichostatin A provides robust neuroprotection against *in vitro* and *in vivo* models of cerebral ischemia, suggesting a role for histone acetylation in ischemic brain injury [7], [11]. Endogenous neuroprotection by ischemic preconditioning, i.e. a sublethal ischemic stimulus which confers increased resistance to a severe ischemic insult, depends on *de novo* expression of neuroprotective genes mediated by transcription factors like HIF-1, CREB or NF-κB [20]. Whether histone acetylation and/or CBP activity play a role for the acquisition of ischemia-tolerant state in neurons, however, is currently unknown. Here we demonstrate that CBP-mediated histone acetylation is crucial for neuronal survival. Further, we tested whether endogenous neuroprotection by ischemic preconditioning is linked to changes in histone acetylation, CBP recruitment and essential for the acquisition of an ischemia-tolerant state in neurons.

## Materials and Methods

### Animals

*In vivo* ischemic injury and preconditioning experiments were performed on male C57BL/6N mice (18–22 g, 8–12 weeks old, Charles River, Germany). Animals were maintained on a 12 h light/dark cycle and given food and water *ad libitum*. They were acclimatized for at least 1 week before surgery. Animals were kept under specific pathogen free (SPF) conditions and regularly screened for infections according to FELASA protocols. All efforts were made to minimize the number and suffering of animals used. The generation of CBP^+/−^ mice has been described previously [21]. The experiments with CBP^+/−^ mice were performed on a DBA and C57BL/6J mixed background, since these mutants are not viable on a pure C57BL/6J background [12]. All experimental procedures were approved by the respective official committees and carried out in accordance with the Animal Welfare Act, the European Communities Council Directive of November 24, 1986 (86/609/EEC) and the ARRIVE (Animals in Research: Reporting *In Vivo* Experiments) guidelines [22].

### Antibodies

The following antibodies were used for immunoblotting, immunocytochemistry or chromatin immunoprecipitation: rabbit anti-acetylated histone-H3 and -H4 from Millipore (Schwalbach/Ts., Germany); rabbit anti-CBP (A-22), goat anti-actin, and rabbit anti-GFP from Santa Cruz (Santa Cruz, CA, USA).

### Primary neuronal cell cultures

Primary neuronal cultures of cerebral cortex were obtained from embryos (E16–E18) of Wistar rats or from embryos (E15–E16) of C57BL/6N or CBP^+/−^ mice. Cultures were prepared and maintained in neurobasal medium with B27 supplement as previously described [23].

### Combined oxygen-glucose deprivation (OGD); Curcumin treatment

In all *in vitro* experiments, serum-free primary neuronal cultures were used after DIV 9. OGD experiments were conducted as previously described [23]. Briefly, culture medium was removed from cells and preserved. Cells were rinsed twice with warmed PBS, placed in OGD chamber (a humidified, temperature-controlled chamber (36±0.5°C) at PO_2_<2 mmHg). PBS was replaced by a balanced salt solution (BSS_0_). OGD was terminated by taking the culture plates out of the OGD chamber and replacing BSS_0_ by conditioned medium (of 50% fresh cultivating medium and 50% preserved cell culture medium). At various time points after OGD, aliquots of the medium were saved for the analysis of cellular death/viability and determined morphologically by phase contrast microscopy. For ischemic preconditioning, the duration of OGD was 30 min, whereas OGD duration for injurious ischemia ranged from 75 min to 150 min. The time interval between ischemic preconditioning stimulus and injurious OGD was 24 h. Curcumin was dissolved in DMSO to give a 10 mM stock solution, diluted in medium to final concentrations of 1–16 µM. In ischemic preconditioning experiments, Curcumin was applied to cortical neuronal cell cultures following preconditioning OGD i.e. 24 h before injurious OGD. Vehicle-treated cultures received 0.01% DMSO in medium.

### Construction, production, and in vitro knockdown efficiency of lentivirus-expressing CBP embedded microRNAs (miR-shRNA)

Third generation lentivirus was generated as described previously [24], [25]. Briefly, small microRNA-embedded hairpin RNA (miR-shRNA) constructs were generated in pcDNA6.2-GW/EmGFP-miR (Invitrogen) along with an EGFP reporter and driven by a neuron-specific synapsin promoter [24]. A non-targeting control microRNA embedded shRNA served as a control designated ‘scrambled’. Three different targeting regions were tested within the open reading frame of murine CREB binding protein (*Crebbp*, NM_001025432) i.e. AGGCAGCAGCCAGCATTGATA (CBP-miR-shRNA-1), TGTGCCCATGCTGGAAATGAA (CBP-miR-shRNA-2) or CTGCCTCAACATCAAACATAA (CBP-miR-shRNA-3). Neuronal cultures were transduced on DIV 3. After 96 h, transduction efficiencies (>95% of neurons) and multiplicity of infection (approximately 5 MOI) were determined and calculated from serial dilutions in neuronal cultures using enhanced green fluorescent protein (EGFP) fluorescence as a reporter.

### Evaluation of cell survival of transduced cultures

Epifluorescence microscopic images were taken on DIV 9 and 10 using EGFP as a reporter for lentiviral gene delivery and miR-shRNA expression as described [24]. In all, 8 regions of interest (ROIs) were preselected per well and repeatedly analyzed over time, maintaining identical settings for all experiments. Enhanced green fluorescent protein-positive cells were counted in a blinded manner and ratios calculated to compare the effects of CBP miR-shRNA expression on survival after OGD-induced cell loss. Each ROI initially contained ∼85±10 cells on DIV 9. In total, an average of 85×8×4×3 = 8,160 cells per condition (ROI×miR-shRNAs×OGD durations) were analyzed before and after OGD for each independent experiment. For visual display of neuronal survival in a particular ROI, emitted fluorescence was pseudocolored green (just before OGD) and red (24 h after OGD) and images were merged. The resulting yellow was indicative of surviving neurons.

### Immunoblots

For total cellular protein extraction, cells or brain tissues were lysed in ristocetin-induced platelet agglutination (RIPA) buffer [50 mm Tris pH 7.4, 150 mm NaCl, 0.1% w/v sodium dodecyl sulphate (SDS), 1% w/v Triton X-100, 1% w/v sodium deoxycholate and protease inhibitor cocktail (Roche)] and clarified at 12000×g for 5 min at 4°C. For extraction of nuclear proteins, cells or brain tissues were lysed in cell lysis (CL) buffer [10 mM HEPES, 2 mM magnesium chloride, 1 mM EDTA, 1 mM EGTA, 10 mM potassium chloride, 1 mM dithiothreitol (DTT), 10 mM sodium fluoride, 0.1 mM sodium vanadate, 1% Nonidet P 40, protease inhibitor cocktail (Roche)] and clarified at 12000×g for 1 min. Pellets were further used for extraction of nuclear proteins in nuclear lysis (NL) buffer [25 mM HEPES, 500 mM sodium chloride, 5 mM magnesium chloride, 10 mM sodium fluoride, 1 mM dithiothreitol (DTT), 10% glycerol, 0.2% Nonidet P 40, protease inhibitor cocktail (Roche)], sonicated (Bandelin, Sonorex Super 10P, Bandelin Electronic, Berlin, Germany) for 1 min at 4°C and clarified at 12000×g for 5 min. Immunoblots were performed as described [23]. Western blotting images were quantified using ImageJ program.

### Immunocytochemistry

Primary cortical cultures were fixed with 4% paraformaldehyde in PBS as described [23] and incubated with primary antibody raised against CBP (diluted 1∶250, Santa Cruz, secondary antibody conjugated with Rhodamine X) and DNA counterstaining with Sytox Green (Invitrogen). Cover slips were mounted using ImmunoFluor Mounting Medium (ICN Biochemicals, Costa Mesa, CA, USA). Images were acquired using a Leica fluorescence microscope and a digital camera.

### Chromatin immunoprecipitation (ChIP) assay

ChIP assay was performed using a kit purchased from Upstate (Lake Placid, NY, USA), according to the manufacturer's procedures and modifications described previously [7]. In short, proteins were formaldehyde cross-linked to chromatin in neurons and cells were harvested, lysed, and the nuclei were sonicated (Bandelin, Sonorex Super 10P, Bandelin Electronic, Berlin, Germany) to shear DNA in lengths between 200 and 1000 base pairs. Following centrifugation, the supernatant was diluted in ChIP dilution buffer and pre-cleared using protein A sepharose slurry containing salmon sperm DNA. Subsequently, the chromatin solution was incubated overnight at 4°C with anti-acetyl histone H4 and CBP antibodies (see above), along with a non-specific rabbit IgG immunoprecipitation. Immune complexes were collected with protein A sepharose, cross-links were reversed for 4 h at 65°C and chromatin-associated proteins were digested by proteinase K. DNA was then extracted with phenol–chloroform, precipitated in ethanol and assayed by quantitative real-time PCR (LightCycler). Thermal cycling started with 10 min at 95°C, followed by 30 cycles of 95°C for 15 s, 68°C for 10 s and 72°C for15 s (amplification product data acquisition at 86°C). For amplification and detection, we used LightCycler Relative Quantification Software (Roche Molecular Biochemicals). We performed a calibration of PCR by a serial dilution of a gelsolin promoter fragment in the range in which we measured the precipitated genomic DNA. In this range, the PCR efficiency (E) for gelsolin promoter genomic DNA was 1.83. For normalization and control, PCR experiments were performed with total (input) DNA and DNA isolated after the precipitation procedure with non-specific rabbit IgG. For quantification, we used the input DNA fraction for normalization and calculated according to the Delta-Cp approach using the expression 1.83– Delta-Cp. The following sequence-specific primers (MWG Biotech, Ebersberg, Germany) were used: Gelsolin promoter forward, 5′-GAACCCAGATGTCTCAGAGAT-3′; Gelsolin promoter reverse, 5′-CCGCGCCTCAGACACCCGAC-3′.

### Quantitative real-time RT-PCR

Total cellular RNA was extracted using Trizol reagent and followed by complementary DNA (cDNA) synthesis. The expression of each sample was normalized for RNA preparation and RT reaction on the basis of its glyceraldehyde-3-phosphate dehydrogenase (GAPDH) mRNA content [26]. For detection of the amplification products in GAPDH and gelsolin RT-PCR, we used a LightCycler-FastStart DNA Master SYBR Green I Kit (Roche Molecular Biochemicals, Penzberg, Germany). Thermal cycling started with 10 min at 95°C, followed by 30 cycles of 95°C for 15 s, 68°C for 10 s and 72°C for 15 s (amplification product data acquisition at 86°C). For amplification and detection, we used LightCycler Relative Quantification Software (Roche Molecular Biochemicals). To determine the RT-PCR efficiency (E), we analyzed a serial dilution of a GAPDH and gelsolin cDNA over the range in which we measured cDNA. In this range, the PCR efficiency (E) for both genes was 1.88. The relative gelsolin to GAPDH expression was calculated using the Delta-Cp approach based on the expression 1.88 – Delta- Cp. The following sequence-specific primers (MWG Biotech, Ebersberg, Germany) were used:

GAPDH forward, 5′-AGATTGTCAGCAATGCATCCTGC-3′;

GAPDH reverse, 5′-CCTTCTTGATGTCATCATACTTGG-3′;

Gelsolin forward, 5′- CAGCCTCTGACTTCATCTCCAAG-3′;

Gelsolin reverse, 5′-CACGTTGGCAATGTGGCTGGAG-3′.

### Lactate dehydrogenase (LDH) assay for assessment of cellular injury

Neuronal injury after OGD was assessed by the measurement of LDH in culture medium in a kinetic photometric assay (at 340 nm) at 24 h after the injury paradigm as described [23].

### Middle cerebral artery occlusion (MCAo) as ischemic injury and preconditioning paradigms *in vivo*

Animal experiments were performed according to institutional and international guidelines. Mice were anesthetized for induction with 1.5% isofluorane and maintained in 1.0% isoflurane in 70% N_2_O and 30% O_2_ using a vaporizer. Ischemia experiments were essentially performed as described [27], [28]. In brief, brain ischemia was induced with an 8.0 nylon monofilament coated with a silicone resin/hardener mixture (Xantopren M Mucosa and Activator NF Optosil Xantopren, Haereus Kulzer, Germany). The filament was introduced into the left internal carotid artery up to the anterior cerebral artery. Thereby, the middle cerebral artery and anterior choroidal arteries were occluded. Filaments were withdrawn after 30 min to allow reperfusion. Regional cerebral blood flow (rCBF) measured using laser-Doppler-flowmetry (Perimed, Jarfälla, Sweden) fell to less than 20% during ischemia and returned to approximately 100% within 5 min after reperfusion in either group (P>0.05). Core temperature during the experiment was maintained at 36.5°C±0.5°C with a feed-back temperature control unit. As a control, sham-operated mice underwent identical surgery but did not have the filament inserted. For ischemic preconditioning, all the surgical procedures were the same except for the duration of occlusion, which was 5 min. The time interval between preconditioning occlusion and injurious occlusion was 24 h.

### Determination of brain lesion size

Animals were sacrificed at 24 h after brain ischemia. Brains were snap-frozen in isopentane for cryostat sectioning. Ischemic lesion size was measured by computer-assisted volumetry of serial 20 µm-thick hematoxylin stained coronal brain sections (2 mm apart) as described in detail previously [29]. Lesion volume was determined by summing up the volumes of each section directly or indirectly using the following formula: contralateral hemisphere (mm^3^) − undamaged ipsilateral hemisphere (mm^3^). The difference between direct and indirect lesion volumes is likely attributable to brain swelling.

### Statistical evaluation

Data were pooled from experiments as indicated in the figure legends and presented as mean ± SEM. For statistical analysis Student's t-test (lesion volumes), ANOVA on ranks (cell viability after OGD in CBP-deficient cultures) test and one-way ANOVA followed by Tukey's post hoc (for all the other data) were utilized as applicable (SigmaSTAT statistical software and GraphPad Prism program).

## Results

### Histone acetylation levels are reduced in cortical neurons after injurious ischemia

**Figure 1:**
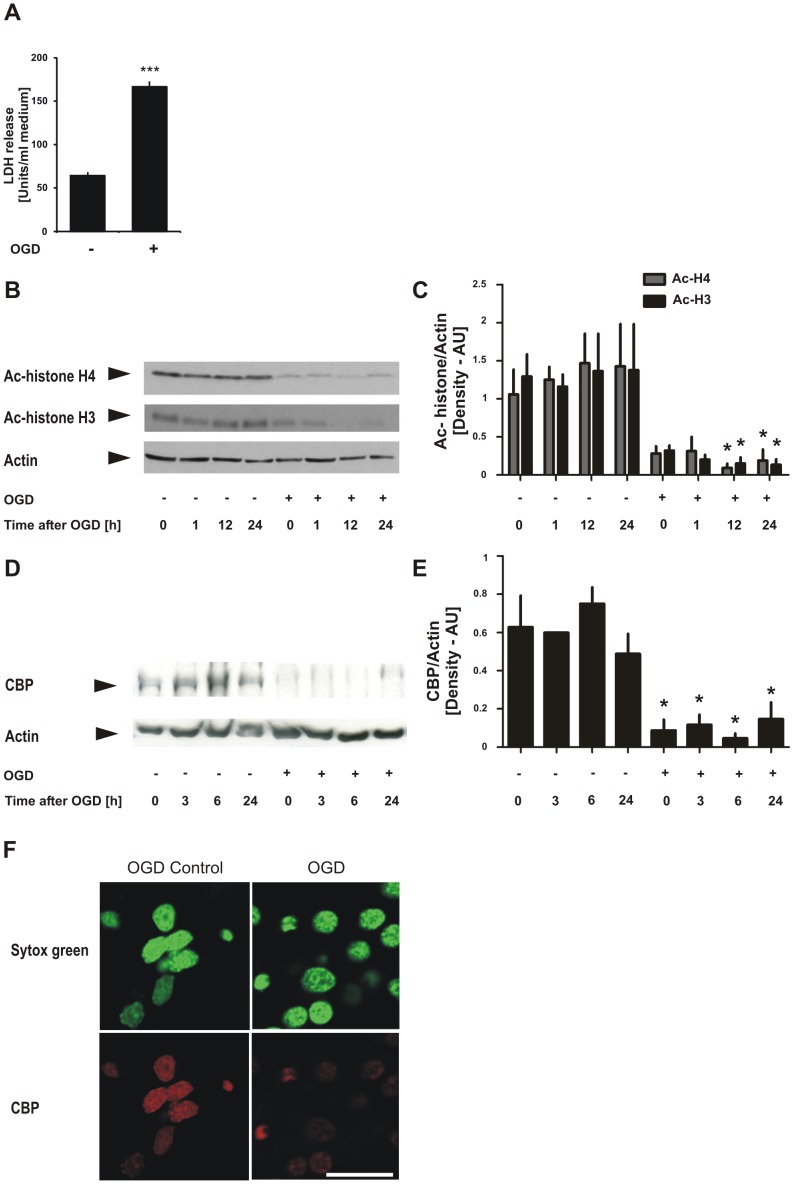
Injurious ischemia in primary cortical cultures induces a strong reduction in histone acetylation and a rapid decrease in CBP protein levels. **A**, Rat cortical neurons were exposed at DIV 9 (*in vitro* day 9) to oxygen-glucose deprivation (OGD) for 150 min; 24 h after the injurious treatment, neuronal death was monitored by determining the release of lactate dehydrogenase (LDH) in culture medium. Bar graphs represent the average LDH release of 4 independent experiments ± SEM. ***p<0.0001. **B**, Representative result of Western blotting analyses of neuronal cultures at various time points following injurious OGD using antibodies against ac-histone H4, ac-histone H4 and actin. **C**, Quantification of Western blotting images was performed using ImageJ program and one-way ANOVA followed by Tukey test was conducted for statistical analyses. Bar graphs represent the mean values from 3 experiments ± SEM. *p<0.05. **D**, Representative result of Western blotting analyses of rat cortical neurons following injurious oxygen-glucose deprivation (OGD) for 150 min at DIV 9 (*in vitro* day 9) using antibodies against CBP and actin. E, Quantification of the Western blotting images was performed using ImageJ program and one-way ANOVA followed by Tukey test was conducted for statistical analyses. Bar graphs represent the mean values from 3 experiments ± SEM. *p<0.05. F, Representative images of immunocytochemical staining of cortical neurons that were fixed immediately after 150 min injurious OGD. Nuclear staining (green), CBP (red). Sytox Green dye was used for DNA counterstaining of nucleic acids. Scale bar, 30 µm. The images are representative results of 3 independent experiments.

To investigate whether acetylation of histones in neurons is affected by ischemia, we exploited an established *in vitro* model of ischemic cell death. In this assay, rat primary cortical neurons were exposed to injurious oxygen-glucose deprivation (OGD) for 150 min on *in vitro* day 9 in culture (DIV 9). At 0, 1, 12 and 24 h after the termination of OGD, proteins were extracted and analyzed by western blotting. The release of lactate dehydrogenase (LDH) into the culture medium was measured 24 h after OGD as an indirect marker of cellular disruption. Figure 1 shows that 150 min OGD, which caused significant damage to neurons as measured by increase in LDH levels (Figure 1A), decreased acetylation levels of both histone H4 and histone H3 throughout all the time points examined reaching statistical significance particularly at 12 and 24 h after OGD (Figure 1B and 1C). In OGD control cultures, histone acetylation levels did not change. This result shows that injurious ischemia causes a reduction in bulk acetylation levels of histone -H4 and -H3 in neurons.

### CREB-binding protein (CBP) level is reduced in cortical neurons after injurious ischemia

Next, we tested whether protein levels of CBP, a pivotal histone acetyltransferase in neurons, were altered in cortical neurons after an ischemic insult. Again, rat primary cortical cultures were subjected to OGD for 150 min at DIV, and subsequently proteins were extracted particularly at early time points, 0, 3, 6, and 24 h after the termination of OGD. Western immunoblotting using an antibody against CBP revealed that, compared to control cultures, CBP protein levels were significantly decreased in neurons at all the time points examined after injurious OGD (Figure 1D and 1E). To gain a more detailed insight into CBP reduction after ischemia, we conducted immunocytochemical staining for CBP using rat primary cortical cultures immediately after their 150 min exposure to injurious OGD. We found that CBP protein signal was decreased in neurons already at the termination of injurious OGD, i.e 0 h after OGD (Figure 1F). This result demonstrates that injurious ischemia leads to rapid decrease in CBP protein levels in neurons.

### CBP^+/−^ neurons are highly sensitive to ischemic injury

**Figure 2:**
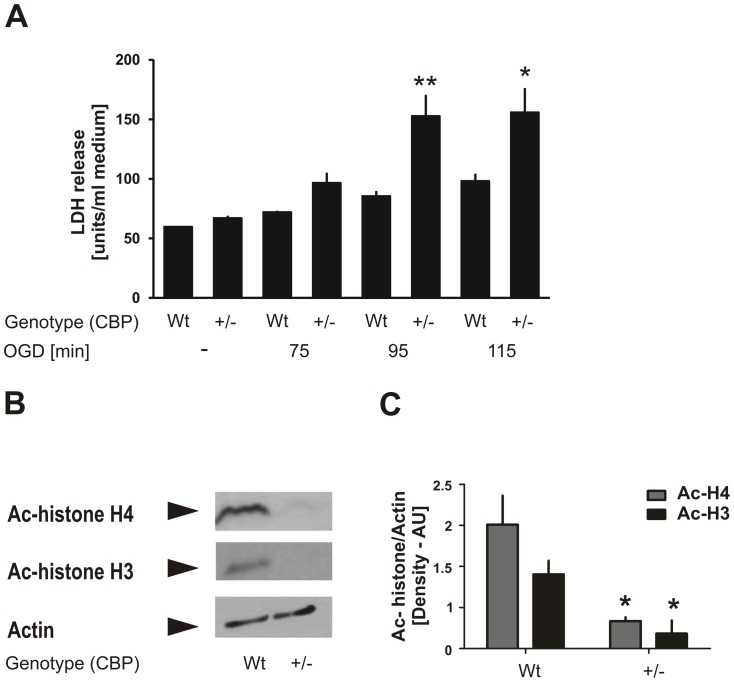
Exacerbation of ischemic injury in CBP^+/−^ primary cortical cultures. **A**, Cortical neurons isolated from CBP^+/−^ mice or wild-type littermates were exposed at DIV 9 (*in vitro* day 9) to oxygen-glucose deprivation (OGD) for 75, 95 or 115 min. Lactate dehydrogenase (LDH) release was measured after 24 h as a marker to quantify neuronal death. Bar graphs represent the average LDH release of 4 independent experiments ± SEM. **p<0.01; *p<0.05. **B**, Representative result of Western blotting analyses of CBP^+/−^ mice and wild-type littermate cortical cultures using antibodies against ac-histone H4, ac-histone H4 and actin. **C**, Quantification of the Western blotting images was performed using ImageJ program and t test was conducted for statistical analyses. Bar graphs represent the mean values from 2 experiments ± SEM. *p<0.05.

To determine CBP's importance for neuronal survival after injurious ischemia, we conducted OGD experiments using cortical neurons from CBP heterozygous knockout (CBP^+/−^) mice. CBP^+/−^ mice are an established model for Rubinstein-Taybi syndrome [21] and a useful tool for studies of CBP and histone acetylation in learning and memory processes [12]. Here, we exposed primary cortical cultures isolated from CBP^+/−^, as well as neurons isolated from wild-type littermate mice, to injurious OGD for different durations ranging from 75 min to 115 min at DIV 9. Lactate dehydrogenase (LDH) release into the culture medium was measured 24 h after OGD as an indirect measurement of cell death. Figure 2A demonstrates that neuronal damage increased with longer durations of OGD, as expected. Yet it was further exacerbated in the CBP^+/−^ cultures, reaching statistical significance particularly after the longest OGD durations of 95 min and 115 min. Similar basal LDH levels in CBP^+/−^ and wild-type cultures in the OGD control condition indicated that cell viability under normal culture conditions did not differ significantly between neurons of the two genotypes. To study whether histone acetylation levels were altered in the CBP^+/−^ neurons under normal conditions, we extracted protein lysates from CBP^+/−^ and wild-type primary cortical cultures and carried out Western immunoblotting using antibodies against acetyl-histone H4 and acetyl-histone H3. Figure 2B and 2C show a significant reduction in acetylation levels of both histone H4 and histone H3 in CBP^+/−^ primary cortical neurons. These results suggest a causal relationship between reduced CBP and histone acetylation levels, and enhanced neuronal vulnerability to ischemic cell death.

### Knock-down of CBP expression enhances neuronal sensitivity to ischemic injury

**Figure 3:**
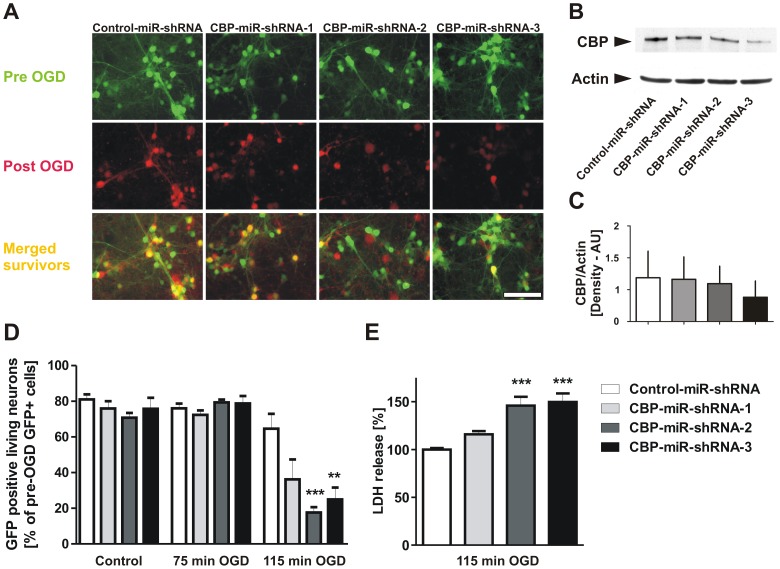
Reduction of CBP in cortical neurons by miR-shRNA treatment exacerbates ischemic injury responses. **A**, Mouse cortical neurons were transduced at DIV 3 (*in vitro* day 3) with lentivirus-expressing CBP-specific miR-shRNA. At DIV 9, cultures were exposed to oxygen-glucose deprivation (OGD). Representative photomicrographs showing GFP expression that were taken shortly before and 24 h after OGD from the same microscopic fields. Scale bar, 100 µm. **B**, Representative result of Western blotting analyses of cortical neurons at DIV 9 after transduction with lentivirus-expressing miR-shRNAs using antibodies against CBP and actin. Reduction in CBP protein levels is particularly observable in cultures transduced with miR-shRNA-2 and miR-shRNA-3. **C**, Quantification of the Western blotting images was performed using ImageJ program and one-way ANOVA followed by Tukey test was conducted for statistical analyses. Bar graphs represent the mean values from 3 experiments ± SEM. **D**, Cortical neurons were transduced with the indicated lentiviral particles and analyzed after 75 and 115 min OGD. Survival of GFP-positive neurons exploited cell counts of pre- and post-OGD photomicrographs with mean data pooled from 3 independent experiments ± SEM. ***p<0.001; **p<0.01. **E**, Cortical neurons were transduced with the indicated miR-shRNAs and subjected to 115 min of injurious oxygen-glucose deprivation (OGD). Neuronal cell death was monitored by the release of lactate dehydrogenase measurement (LDH) from 3 independent experiments ± SEM. ***p<0.001.

In order to pinpoint CBP's role for neuronal survival, excluding its other possible effects, e.g. on neuronal development, we generated miR-shRNAs specifically directed against CBP mRNA in a lentiviral knockdown system [24]. Three CBP-specific miR-shRNAs, each of which target CBP transcript at a different position, and a nontargeting control miR-shRNA were delivered into mouse primary cortical cultures at DIV 3 by lentiviral transduction. Pre-OGD photomicrography on DIV 9 demonstrated high GFP expression in neurons from all four conditions, ensuring the high efficiency of lentiviral infections (Figure 3A). To validate knockdown of CBP expression by miR-shRNAs, proteins were extracted at DIV 9 and western immunoblotting was carried out using antibodies against CBP, and actin. Figure 3B and 3C show reduced CBP expression in neurons by all target specific miR-shRNAs yet CBP-miR-shRNA-3 seemed to be the most effective miR-shRNA candidate in reducing CBP protein levels. To characterize the functional consequences of RNA interference directed against CBP transcript for neurons after ischemic injury, we exposed mouse primary cortical cultures to 75 or 115 min OGD on DIV 9, i.e. six days after infection with lentiviruses expressing miR-shRNAs. After 24 h OGD, neuronal injury was assessed by GFP-positive cell counts and LDH assay. When compared to infection with lentivirus-expressing nontargeting miR-shRNA or non-infected naïve neurons (data not shown), infection with lentivirus-expressing CBP-specific miR-shRNAs did not affect basal neuronal viability before OGD or under OGD control conditions. However, CBP-specific miR-shRNAs sensitized cortical neurons to ischemia-induced cell death, as the number of GFP-positive living neurons was decreased by all three miR-shRNAs, in particular by CBP-miR-shRNA-2 and CBP-miR-shRNA-3, at 24 h after injurious OGD for 115 min (Figure 3D). This finding was further reinforced by LDH measurements demonstrating that neuronal death was exacerbated by CBP-specific miR-shRNAs and that miR-shRNA-2 and miR-shRNA-3 enhanced the ischemic damage significantly when assessed 24 h after severe OGD (Figure 3E). In summary, these results confirm the causal link between the reduction in CBP expression levels and increased neuronal sensitivity to ischemic injury.

### Ischemic preconditioning alters neuronal histone acetylation levels *in vitro*

**Figure 4:**
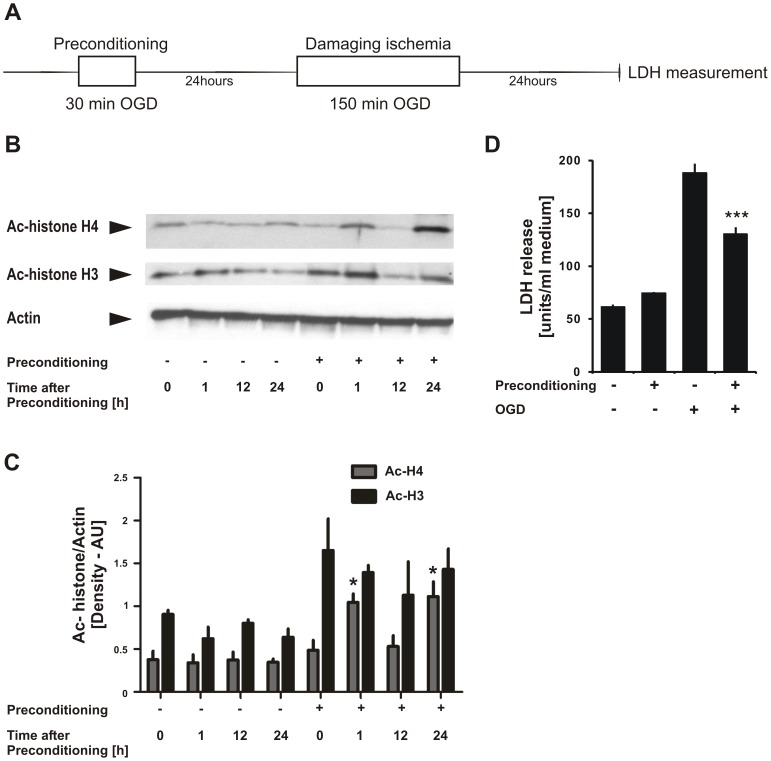
Changes in histone acetylation in cortical neurons after ischemic preconditioning. **A**, Experimental design. Rat cortical neurons were exposed to 30 min non-injurious preconditioning oxygen-glucose deprivation (OGD) on DIV 9 (*in vitro* day 9) and, 24 h afterwards, subjected to 150 min injurious damaging OGD. Neuronal cell death was monitored by lactate dehydrogenase analysis (LDH) further 24 h later. **B**, Representative result of Western blotting analyses of neuronal cultures at various time points after preconditioning using antibodies against ac-histone H4, ac-histone H3 and actin. **C**, Quantification of the Western blotting images was performed using ImageJ program and one-way ANOVA followed by Tukey test was conducted for statistical analyses. Bar graphs represent the mean values from 4 experiments ± SEM. *p<0.05. **D**, Neuronal death was assessed by LDH measurement as a marker for neuronal death in preconditioned and non-preconditioned cultures after their exposure to 150 min injurious OGD. Thirty min preconditioning OGD reduced LDH levels significantly, providing protection against 150 min injurious OGD. Bar graphs represent the average LDH release of 4 independent experiments ± SEM. *p<0.001.

We have demonstrated that CBP expression is crucial for the outcome after injurious OGD and that it determines the threshold for neuronal survival after ischemic injury *in vitro*. To test the hypothesis that histone acetylation status and CBP protein expression is linked to mechanisms of endogenous neuroprotection, we applied an *in vitro* model for ischemic preconditioning: Rat primary cortical cultures were subjected to a short (30 min) episode of OGD that is not injurious for neurons as a preconditioning stimulus. After an interval of 24 h, preconditioned as well as non-preconditioned control cultures were exposed to prolonged OGD (150 min). Twenty-four hours after this injurious ischemic insult neuronal death was assessed in a lactate dehydrogenase (LDH) assay (Figure 4A). Figure 4D shows the resulting neuronal damage as detected by increase in LDH release. The injury was significantly reduced in those cultures that had previously undergone preconditioning OGD. Ischemic preconditioning by itself did not increase LDH release as measured prior to injurious OGD. To assess whether histone acetylation was altered in neurons after preconditioning, we extracted protein lysates from rat primary cortical cultures at 0, 1, 12 and 24 h after their exposure to 30 min preconditioning OGD. Acetylation of histone H4 as well as histone H3 was determined by western immunoblotting. Figure 4B and 4C suggests a dynamic alteration of histone H4 and histone H3 acetylation levels in neurons after their exposure to the 30 min preconditioning OGD. Histone H4 acetylation was significantly increased at 1 h and 24 h, whereas enhanced histone H3 acetylation was detected already at the initiation of re-oxygenation (0 h) as well as at 1 h and 24 h after the preconditioning OGD. This result indicates that acetylation levels of histone -H4 in particular and -H3 are altered by ischemic preconditioning in neurons *in vitro*.

### Curcumin, a CBP HAT activity inhibitor, attenuates ischemic preconditioning in primary cortical cultures

**Figure 5:**
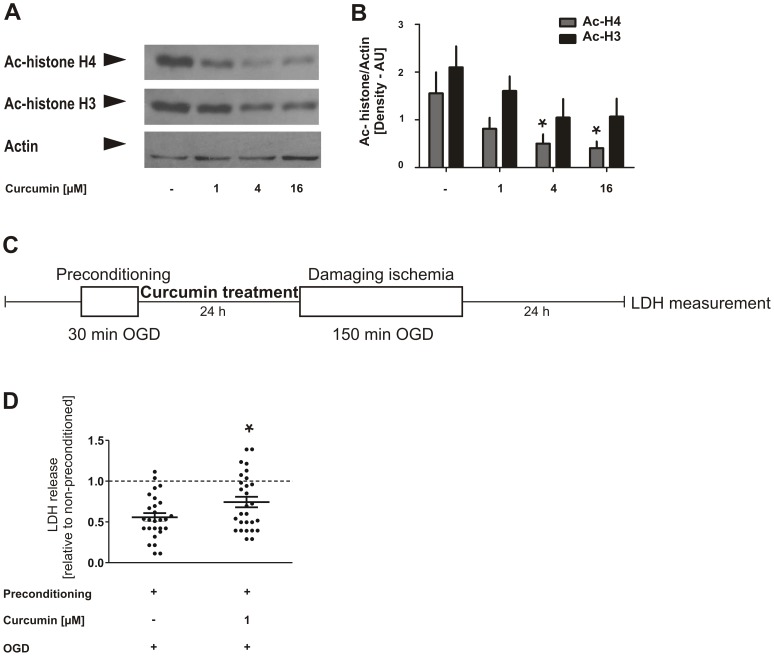
Curcumin, a CBP HAT activity inhibitor, reduces histone acetylation and attenuates ischemic preconditioning in primary cortical cultures. **A**, Representative result of Western blotting analyses of neuronal cultures following treatment with indicated concentrations of Curcumin for 24 h using antibodies against ac-histone H4, ac-histone H4 and actin. **B**, Quantification of the Western blotting images was performed using ImageJ program and one-way ANOVA followed by Tukey test was conducted for statistical analyses. Bar graphs represent the mean values from 4 experiments ± SEM. *p<0.05. **C**, Experimental design. Rat cortical neurons were exposed to 30 min non-injurious preconditioning oxygen-glucose deprivation (OGD) on DIV 9 (*in vitro* day 9), and treated with 1 µM curcumin for the 24 h interval between the preconditioning and injurious OGDs. Damaging OGD of 150 min was conducted on DIV 10, and neuronal cell death was monitored by lactate dehydrogenase analysis (LDH) further 24 h later. **D**, Neuronal death was assessed 24 h after the injurious OGD exploiting the increase in LDH in the culture medium. Curcumin per se had no significant impact on the release of LDH in controls or OGD treated cultures which were not exposed to preceding preconditioning OGD. *p<0.05, unpaired t-test. The graph shows LDH release in preconditioned cultures relative to non-preconditioned cultures for vehicle and curcumin treatment conditions. N = 2 experiments. Data is shown as scattered dot blots representing cell culture wells with mean relative protection ± SEM.

Having demonstrated the concurrence of increased acetylation status with an increased resistance of neurons to ischemic stress after ischemic preconditioning, we wanted to test whether pharmacological inhibition of HAT activity interferes with the epigenetic reprogramming necessary to restore neuron resilience after ischemic preconditioning. For this purpose, we utilized curcumin, an inhibitor of CBP's HAT activity [30]. Firstly, to demonstrate that curcumin has indeed the expected effect on histone acetylation levels, we treated rat primary cortical cultures with curcumin at doses of 1, 4 and 16 µM for 24 h, extracted nuclear proteins and carried out western immunoblotting using antibodies against acetyl-histone H4 and acetyl-histone H3. Curcumin reduced acetylation levels of both histone H4 in particular and histone H3 in a dose-dependent manner (Figure 5A and 5B). We then tested curcumin's effects on ischemic preconditioning and exposed rat primary cortical cultures to 30 min preconditioning OGD. We directly treated the cultures with 1 µM curcumin until they were exposed to injurious OGD 24 h afterwards. Measurement of lactate dehydrogenase (LDH) release into the culture medium was carried out to assess cell death 24 h after the injurious OGD (Figure 5C). Curcumin treatment, although applied only during the interval between preconditioning OGD and injurious OGD, partially prevented the reduction of LDH release by ischemic preconditioning, i.e. curcumin significantly attenuated the induction of ischemia-tolerant state in neurons (Figure 5D). This finding suggests that CBP's HAT activity, at least in part, plays an important role in induction of the ischemia-tolerant state in neurons.

### Ischemic preconditioning results in early increase in histone H3 acetylation levels in mouse brain

**Figure 6:**
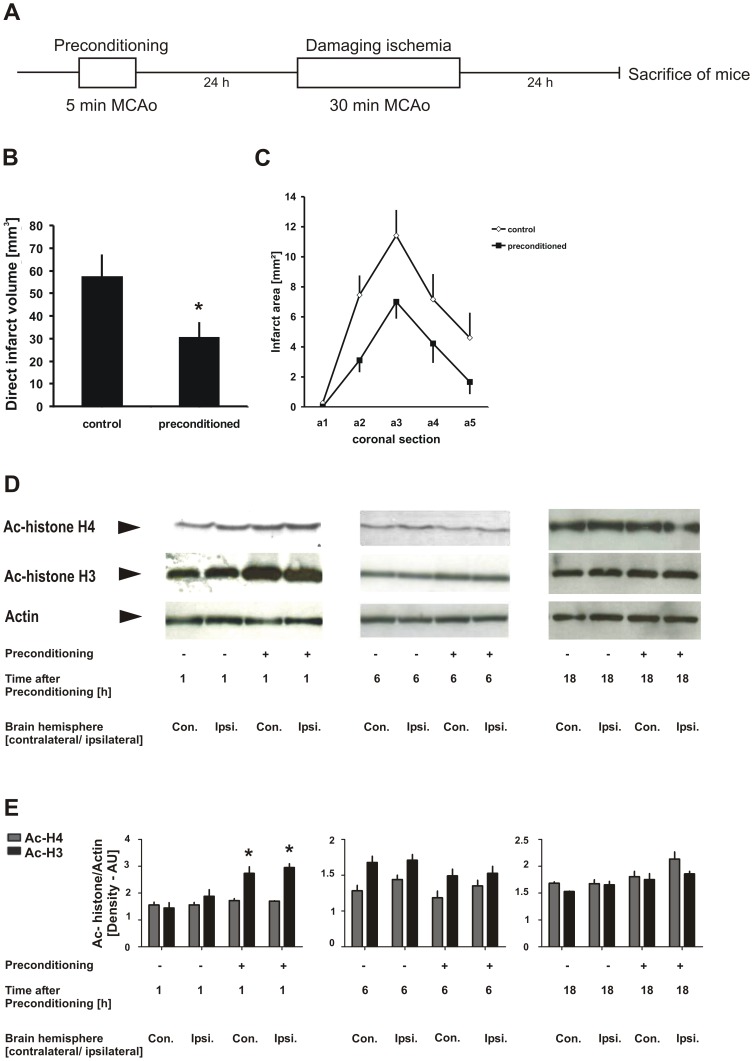
Early increase in Histone H3 acetylation levels in mouse brains after ischemic preconditioning. **A**, Experimental design. C57BL/6N mice underwent 5 min preconditioning filamentous middle cerebral artery occlusion (MCAo) followed by 30 min injurious MCAo of the same artery, with a 24 h interval. Mice were sacrificed at 24 h after the 30 min injurious MCAo, followed by quantitative assessment of brain damage by computer-assisted infarct volumetry using 20 µm thick, hematoxylin-stained brain sections. **B, C**, Results of brain infarct size assessment as total volume (B), and as areas in five anterior-posterior coronal sections (C). Preconditioning MCAo conferred a significant reduction in brain infarct volume after 30 min injurious MCAo. Student's t-test; *p<0.05. Data are presented as means ± SEM. **D,F** Brain homogenates of both hemispheres were analyzed by western blotting with the indicated antibodies at 1 h, 6 h and 18 h of 5 min preconditioning MCAo or sham preconditioning. Immunoblot images (D) are representative of three independent biological replicates (i.e. analysis of 6 animals per time point; analysis of a total number of 18 animals; no animals were excluded). **E**, Quantification of the Western blotting images was performed using ImageJ software. Bar graphs represent mean values ± SEM from 3 biological replicates (3 preconditioned and 3 sham-preconditioned mice per time point). *p<0.05, one-way ANOVA followed by Tukey's post hoc test.

Next, we aimed to extend our *in vitro* findings to an *in vivo* mouse model of ischemic preconditioning. C57BL/6N mice underwent a non-injurious filamentous occlusion of the left middle cerebral artery (MCAo) for 5 min, as a preconditioning stimulus (preconditioning MCAo; n = 19), or a sham operation (sham preconditioning; n = 16). Following an interval of 24 h, the middle cerebral artery was occluded for a full 30 minutes (Figure 6A). Animals were sacrificed after an additional reperfusion period of 24 h. Brains were snap-frozen and cerebral lesion volume was determined by computer-assisted volumetry on serial coronal sections as described in detail previously [29]. The 5-min preconditioning MCAo effected a significant reduction in the infarct volumes resulting from 30 min MCAo (Figure 6B). One animal per group died, so the histological analysis is based on 18 animals [preconditioning MCAo group] and 15 animals [sham preconditioning group]. Cerebral infarct areas in all anterior to posterior coronal brain sections were smaller in preconditioned mice (Figure 6C). Next, histone acetylation changes caused by ischemic preconditioning were studied at reperfusion intervals of 1 h, 6 h and 18 h after the 5 min preconditioning MCAo. Whole hemisphere protein extracts were analyzed by western blotting and probed with antibodies against acetylated histone H4 and histone H3. Significantly increased histone H3 acetylation levels were observed 1 h after preconditioning MCAo in the ipsilateral hemispheres (i.e. the hemisphere on which 5 min MCAo had been performed), and, interestingly, also in the contralateral hemisphere which had not been subjected to MCAo (Figure 6D and 6E). By contrast, preconditioning did not impact the acetylation status of histone H4. The specific increase of H3 acetylation early after ischemic preconditioning was not sustained, since H3 and H4 acetylation levels were not altered at 6 h or 18 h following the 5 min preconditioning MCAo (Figure 6D and 6E). In summary, the acetylation level of histone H3 is significantly increased in both hemispheres of the mouse brain 1 h after focal ischemic preconditioning in the MCA territory *in vivo*.

### Ischemic preconditioning enhances CBP recruitment and histone acetylation levels at the *gelsolin* promoter locus and upregulates *gelsolin* mRNA expression in neurons

**Figure 7:**
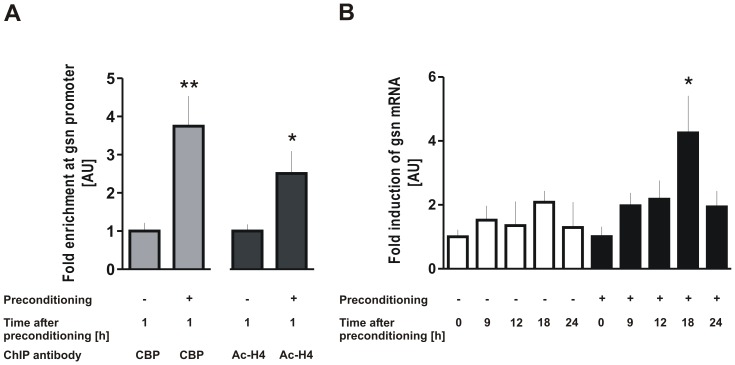
Increased histone H4 acetylation and recruitment of CBP to gelsolin promoter region precedes transcriptional upregulation of gelsolin after ischemic preconditioning. **A**, Rat cortical neurons were subjected to 30 min non-injurious preconditioning OGD and analyzed by chromatin immunoprecipitation (ChIP) with the indicated antibodies after 1 h interval following the preconditioning. Real time PCR results for gelsolin promoter were presented as mean fold enrichment ± SEM. **p<0.01; *p<0.05. N = 4 experiments. **B**, Rat cortical neurons were subjected to 30 min non-injurious preconditioning OGD or control treatment and analyzed by semi-quantitative real-time RT-PCR for gelsolin at the indicated time points. The data is shown as mean ± SEM. *p<0.05 versus vehicle. N = 3 experiments.

After observing changes in bulk histone acetylation levels in brain ischemic preconditioning models, we investigated whether such changes could also be detected at specific gene loci in neurons after ischemic preconditioning. It is well established that the delayed protection afforded by ischemic preconditioning in the brain depends on the reprogramming of the transcription of genes. Recent studies have shown a plethora of transcriptional and non-transcriptional mechanisms of endogenous neuroprotection [20]. Here, we focused on epigenetic mechanisms and decided to investigate the promoter region of gelsolin, a neuroprotective gene with well-characterized anti-apoptotic and anti-excitotoxic properties [23], [27]. Using chromatin immunoprecipitation (ChIP), we studied CBP recruitment and histone acetylation levels at the gelsolin promoter region in neurons after ischemic preconditioning. Our western immunoblotting results showed an increase in histone acetylation levels at 1 h after the preconditioning stimuli in neurons and mouse brain (Figure 4B, 4C, 6D and 6E). Accordingly, we isolated chromatin from rat cortical neurons at 1 h after the 30 min preconditioning OGD and conducted ChIP assay using anti-CBP and anti-acetyl-histone H4 antibodies. Resulting immunoprecipitated DNA served as template for quantitative real-time PCR experiments with primers encompassing the gelsolin promoter region (position -153 to 150 into the first exon). CBP recruitment and acetylated histone H4 levels were significantly increased at the gelsolin promoter region in neurons after their exposure to ischemic preconditioning, by more than three-fold and two-fold, respectively (Figure 7A). As CBP recruitment and increase in histone acetylation at regulatory genomic loci are expected to activate transcription, we next measured gelsolin mRNA levels in neurons after ischemic preconditioning. As shown in Figure 7B, quantitative real-time RT-PCR demonstrated that gelsolin mRNA levels were significantly upregulated in rat primary cultures 18 h after the 30 min preconditioning OGD. Although a slight induction of gelsolin mRNA was observed in control cultures, significant upregulation was specifically detected in cultures that underwent ischemic preconditioning. These results suggest that CBP and histone acetylation are likely parts of neuroprotective mechanisms that are induced by ischemic preconditioning in neurons.

## Discussion

Impaired histone acetylation and CBP function have been implicated in cell death in neurodegenerative diseases [18]. Yet, little is known about their involvement in neuronal survival after ischemic injury, and no study to date has examined the role of histone acetylation in ischemic preconditioning in the brain. Our study rendered the following four major findings: i) CBP protein loss and decrease of histone acetylation levels occur coordinately after ischemia and determine the threshold of neuron vulnerability to ischemic injury; ii) Histone acetylation is involved in endogenous neuroprotection both *in vitro* and *in vivo*; iii) Inhibition of CBP's HAT activity attenuates ischemic preconditioning in neurons, and iv) Ischemic preconditioning i.e. endogenous neuroprotection is linked to epigenetic trans-activation via CBP recruitment and histone acetylation at the neuroprotective gene promoter gelsolin followed by gelsolin upregulation *in vitro*. In summary, our study provides novel insights into the role of CBP and histone acetylation in conferring neuronal resilience to ischemia and in endogenous neuroprotective mechanisms underlying ischemic preconditioning. CBP appears to be a promising target for neuroprotective strategies.

### Ischemic injury leads to reduced histone acetylation and CBP protein levels in neurons

CBP loss of function was reported to play a critical role in the pathogenesis of several neurodegenerative conditions including polyglutamine diseases [31], [32], spinocerebellar ataxia type 7 [33], and spinal and bulbar muscular atrophy [34]. In an apoptotic model of primary neurons, CBP was degraded by apoptotic caspases decreasing histone acetylation levels [35]. In primary neuronal cultures transfected with mutant Huntingtin gene, cell toxicity was accompanied by CBP depletion and histone hypo-acetylation [32]. Here, we demonstrate reduced histone acetylation and lower CBP protein levels in neurons after ischemic injury (Figure 1 and 2). It is currently unknown whether and to what extent caspases or proteasomal degradation impact on the loss of CBP after OGD. Our data is consistent with previous reports showing reduction in histone acetylation levels in mouse brain after ischemic brain injury and in a global ischemia model [2], [8]. Involvement of any HAT or HDAC enzyme was not investigated in these studies. Although we lack direct evidence for linking reduction of histone acetylation to loss of CBP, the coordinated changes in the levels of acetylated histones and CBP in our experiments suggest that these two events are likely associated. In keeping with this, reports from various *cbp* knockout mouse models demonstrate decreased neuronal histone acetylation in these mice, underscoring CBP's importance for acetylating histones in neurons [12], [13], [14], [36]. Together with our immunocytochemistry result showing that CBP expression is barely detectable in neurons after OGD (Figure 1F), these findings suggest a strong link between loss of CBP, reduction in histone acetylation and neuronal death after ischemic injury.

### Reduction of CBP expression exacerbates neuronal vulnerability to ischemic injury

Next, we explored the role of reduced histone acetylation and CBP levels in neuronal death after ischemia. Various mouse models have so far been developed and used to study the functions of CBP in the normal brain [12], [13], [21], [36]. For our purpose, we used a CBP heterozygous mutant mouse (CBP^+/−^). Our results from primary neuronal cultures from the heterozygous mice showed a clear loss of histone acetylation levels under normal conditions, and upon exposure to ischemic stress they displayed increased vulnerability, compared to cultures of wildtype littermates. Interestingly, CBP gene dose reduction did not decrease neuronal survival at baseline in our *in vitro* system pointing to a specific role of CBP in disease related ischemic stress. Congruently, several recent publications reported a lack of neuronal death in various CBP loss-of-function mouse models as confirmed by the absence of neurodegeneration in Rubinstein-Taybi syndrome patients [13], [14]. Our findings indicate that although CBP might not be required for neuronal survival under normal conditions, it is essential in preserving cell viability after OGD. Partial elimination of CBP decreased the resistance of neurons to ischemic stress but did not affect their viability at normal conditions. This notion has been also reported in another system related to ischemia: The posttranslational modification system with small ubiquitin-like modifier protein SUMO2/3 was not essential in the physiological state of neurons, but its loss of function, if reduced by RNA interference, significantly exacerbated neuronal vulnerability upon ischemia-like stress [24]. Moreover, in an effort to exclude possible compensatory changes in neuronal cultures of heterozygous CBP mice, we developed a second approach namely by creating/inducing RNA interference against CBP in neurons. Results from these experiments further reinforced the causal link between the reduced CBP protein levels and the increased susceptibility of neurons to ischemic stress after RNA interference with CBP compared to control miR-shRNA expressing cultures. Altogether, our findings indicate a critical role for CBP in neuronal survival after ischemic injury.

### Ischemic preconditioning enhances bulk histone acetylation levels, induces CBP recruitment and histone acetylation at gelsolin promoter followed by gelsolin upregulation and is attenuated by inhibition of CBP's HAT activity

Many different mechanisms have been reported to be involved in the development of brain ischemic preconditioning, including induction of neuroprotective gene expression [20]. Little is known, however, about the role of epigenetic changes in preconditioning-induced neuroprotection. We found that bulk levels of acetylated histones are subject to dynamic changes in two different models of ischemic preconditioning, *in vitro* and *in vivo*. Our near-pure neuronal culture system, with less than 10% astroglial contamination, indicated that neuroprotection achieved in our model was probably due to intrinsic neuronal properties. On the other hand, in our *in vivo* model histone acetylation levels were strikingly increased in both ipsilateral and contralateral hemispheres after the preconditioning MCAo, implying that systemic mechanisms are operative in our *in vivo* model. In support of this notion, bilateral ischemia tolerance against global brain ischemia was achieved in rats in a unilateral forebrain ischemic preconditioning model [37]. Addressing this intriguing phenomenon in our *in vivo* model remains a challenge for further investigations.

*In vitro*, the minimum interval of 24 h required for the acquisition of ischemia-tolerant state underpins the *de novo* gene expression-dependent, delayed pattern of neuronal ischemic preconditioning in our model. At the level of transcriptional regulation, evidence has strongly suggested that transcription factors such as hypoxia inducible factor (HIF), cAMP response element binding protein (CREB) and nuclear factor κB (NF-κB) were driving the expression of neuroprotective genes for the acquisition of ischemic tolerance [38], [39]. Interestingly, genome-wide approaches showed preconditioning-induced fundamental reprogramming of the transcriptional response to ischemic injury, ultimately conferring a neuroprotective phenotype [40], [41]. Our present work provides strong evidence for involvement of histone acetylation and CBP in brain ischemic preconditioning. While involvement of histone acetylation in brain ischemic preconditioning was not reported previously, CBP was demonstrated to be associated with the promoter region of a neuroprotective gene, i.e. bcl-2 in neurons after preconditioning: [42] Strikingly, it was not the binding of CREB, but of CBP to the bcl-2 CRE site that increased after preconditioning ischemia, and blocking CBP binding to the bcl-2 CRE with U0126 (a kinase inhibitor) reduced bcl-2 expression and abrogated ischemic tolerance. Here, we demonstrate that CBP is recruited to the promoter of gelsolin after ischemic preconditioning, a gene product which has potent neuroprotective properties [23], [27]. This presence correlates with enhanced levels of acetylated histone H4 at gelsolin promoter. We then found gelsolin mRNA to be upregulated in neurons after ischemic preconditioning. Moreover, Curcumin, a CBP's HAT activity inhibitor, attenuated ischemic preconditioning-induced ischemia-tolerant state in neurons. Collectively, our findings suggest that histone acetylation enhancement at specific neuroprotective gene promoters together with increased CBP recruitment are likely crucial components of the endogenous neuroprotective mechanism of ischemic preconditioning in brain.
